# The effect of the cholinergic anti-inflammatory pathway on collagen-induced arthritis involves the modulation of dendritic cell differentiation

**DOI:** 10.1186/s13075-018-1759-9

**Published:** 2018-11-28

**Authors:** Di Liu, Tong Li, Hui Luo, Xiaoxia Zuo, Sijia Liu, Shiyao Wu

**Affiliations:** Department of Rheumatology and Immunology, Xiangya Hospital, Central South University, Hunan Province Changsha, 410008 People’s Republic of China

**Keywords:** Rheumatoid arthritis, Dendritic cells, Cholinergic anti-inflammatory pathway, GTS-21

## Abstract

**Background:**

The cholinergic anti-inflammatory pathway (CAP) has a strong anti-inflammatory effect on collagen-induced arthritis (CIA), a classic animal model of rheumatoid arthritis (RA). However, the underlying immune regulatory mechanism remains unclear. Here, we investigated the effect of the CAP on arthritis development and the involvement of dendritic cells (DCs).

**Methods:**

Forty DBA/1 mice were randomly divided into five groups: a control group (sham vagotomy+ phosphate-buffered saline; shamVGX+PBS), a CIA group (shamVGX+CIA + PBS), a vagotomy group (VGX + CIA + PBS), a GTS-21 (4 mg/kg) group (shamVGX+CIA + GTS-4), and a GTS-21 (8 mg/kg) group (shamVGX+CIA + GTS-8). The vagotomy group underwent left cervical vagotomy 4 days before arthritis induction, whereas the sham-vagotomy group underwent vagus nerve exposure. Mice were pretreated with GTS-21 by intraperitoneal injection on the day of surgery. The degree of arthritis was measured by using the arthritis score, hematoxylin and eosin staining, and TRAP (tartrate-resistant acid phosphatase) staining. Flow cytometry was used to detect the expression of CD80 and major histocompatibility complex II (MHC II) on CD11c^+^ DCs in the spleen. Luminex was used to detect the serum concentration of interleukin-6 (IL-6), tumor necrosis factor-alpha (TNFα), and IL-10. Immunohistochemistry was used to detect CD11c expression in the synovium. The effects of GTS-21 on DC differentiation and maturation were examined *in vitro* by treating bone marrow–derived DCs with GTS-21 and assessing differentiation and maturation. Flow cytometry was used to analyze CD80 and MHC II expression on the surface of DCs.

**Results:**

GTS-21 treatment ameliorated clinical arthritis in a mouse model of CIA *in vivo*, decreasing the secretion of pro-inflammatory cytokines in the serum and downregulating CD80 and MHC II expression on DCs in the spleen of CIA mice. GTS-21 treatment strongly suppressed the infiltration of DCs into the synovium. Vagotomy itself did not exacerbate the severity of arthritis in CIA mice. *In vitro*, GTS-21 (10 μmol/L) significantly downregulated CD80 and MHC II in bone marrow–derived immature DCs and this effect was blocked by the α7-nicotinic acetylcholine receptor antagonist methyllycaconitine (MLA). However, GTS-21 had no effects on mature DCs.

**Conclusions:**

The present study provides new insight into the mechanism underlying the effects of the CAP on RA and indicates that the immunosuppressive effect of GTS-21 may be mediated by the inhibition of DC differentiation.

**Electronic supplementary material:**

The online version of this article (10.1186/s13075-018-1759-9) contains supplementary material, which is available to authorized users.

## Introduction

Rheumatoid arthritis (RA) is a chronic autoimmune disease characterized by synovial inflammation and cartilage and bone destruction. Although the pathogenesis is unknown, it is believed that inflammatory cell infiltration into the synovium is the main cause of persisting synovitis [1]. Dendritic cells (DCs) are important innate immune cells and professional antigen-presenting cells that play an important role in immunologic priming. DCs contribute to the pathogenesis of RA. Mature DCs in the synovium and secondary lymphoid organs can present antigens to naïve T cells and induce T-cell activation [2, 3]; DCs secrete inflammatory cytokines such as interleukin-12 (IL-12), IL-23, IL-6, tumor necrosis factor-alpha (TNF-α), and IL-1 to induce Th1, Th2, and Th17 differentiation, aggravating the inflammation of the synovium [4–7]. In addition, DCs produce B cell–activating factor, thus promoting the proliferation of antibody-producing B cells [8]. Studies indicate that DC-based immunotherapy may be effective for the treatment of RA. In a mouse model of collagen-induced arthritis (CIA), DCs with tolerogenic characteristics suppressed the progression of established CIA [9–11]. DC differentiation into a tolerogenic state is an attractive tool to restore self-tolerance in RA and other autoimmune disorders [12]. Moreover, clinical trials confirmed that the strategy is safe, feasible, and acceptable [13, 14]. These data suggest that DCs are a promising target for the treatment of RA.

The cholinergic anti-inflammatory pathway (CAP) is an endogenous anti-inflammatory pathway that links the nervous system and the immune system via the vagus nerve [15]. Activation of the CAP can be achieved by vagus nerve stimulation or cholinergic agonists [16]. GTS-21, a classic cholinergic agonist, can bind to the α7 subunit of the nicotinic acetylcholine receptor (α7nAChR) of inflammatory cells to induce an anti-inflammatory response [17]. A previous study showed that the CAP is involved in the reduction of inflammation in experimental sepsis, acute lung injury, ischemia/reperfusion injury, and pancreatitis [18].

Accumulating evidence indicates that the CAP suppresses inflammation in RA. Stimulation of nicotinic acetylcholine receptors attenuates CIA in mice, whereas knockdown of the α7nAChR aggravates CIA [19]. Previous work from our group confirmed that the CAP inhibits arthritis development by regulating immune cells, such suppressing the TNF-α–dependent inflammatory pathway in synoviocytes and Th1 cells, Th17 cell differentiation, and macrophage migration [20–24]. The α7nAChR is also expressed on the surface of DCs [25]. However, the effects of CAP on DCs remain unclear in RA. Here, we investigated the anti-inflammatory effect of the cholinergic agonist GTS-21 on RA and examined the role of DCs.

## Materials and methods

### Animals

Five-week-old C57BL/6 J male mice and 5-week-old DBA/1 male mice were purchased from the Shanghai Institute of Experimental Animals. The mice were fed adaptively for 1 week. The experiment was approved by the Ethics Committee for Laboratory Animals of Central South University.

### Experimental groups

Forty DBA/1 male mice were randomly divided into five groups: a control group (sham vagotomy + phosphate-buffered saline, sham VGX + PBS), a model group (sham VGX + PBS + CIA), a vagotomy group (VGX + PBS + CIA), a GTS-21 4 mg/kg group (sham VGX + GTS-21 4 mg/kg + CIA), and a GTS-21 8 mg/kg group (sham VGX + GTS-21 8 mg/kg + CIA). To block the CAP, mice in the vagotomy group underwent left cervical vagotomy 4 days before CIA induction, whereas the left vagus nerve was exposed but not cut in the sham operation group. To activate the CAP, mice received different doses of GTS-21 (4 and 8 mg/kg) from 4 days before the induction of CIA to 45 days after the first immunization, and the other group received an equal volume of phosphate buffer by intraperitoneal injection at the same time points.

### CIA induction

Bovine type II collagen (Chondrex, Redmond, WA, USA) was diluted to 2 mg/mL with 10 mM acetic acid and emulsified with an equal volume of Freund’s complete adjuvant (Chondrex). The CIA model mice were injected with 0.1 mL of fully emulsified bovine collagen intradermally at the tail base. At 21 days after the first immunization, CIA mice were injected with the same amount of bovine type II collagen emulsified with Freund’s incomplete adjuvant (Chondrex) as the second immunization.

### Evaluation of arthritis

Starting on the day of the second immunization, each digit and paw were assessed every 3 days by two investigators until day 45. The arthritis index scoring criteria were as follows: 0, normal; 1, slight redness or swelling of the joint; 2, moderate swelling; 3, obvious swelling; and 4, severe swelling and inability to bear weight.

### Histopathological examination and immunohistochemistry

Mice were sacrificed on day 45. The joints were removed, fixed in formalin for 48 h, decalcified in 10% ethylenediaminetetraacetic acid, and embedded in paraffin. The sections were stained with hematoxylin and eosin (HE) and tartrate-resistant acid phosphatase (TRAP) for histopathological analysis. The severity of inflammatory cell infiltration (grade 0–4) and synovial hyperplasia (grade 0–3) were scored as previously described in joints of ankle and knee [26, 27]. TRAP^+^ osteoclasts were counted manually in joints of the ankle and knee. The CD11c expression level in the synovium was examined with immunohistochemistry by using a rabbit anti-mouse CD11c antibody (1:200; Servicebio, Wuhan, China) in accordance with the instructions of the manufacturer. The integrated optical density (IOD) values of tissue sections in each group were measured by Image-Pro Plus 6.0 software (Media Cybernetics, Inc., Rockville, MD, USA) after tissue images were captured under an optical microscope (100×). Five views were randomly selected to determine the positive IOD values and the mean of these values was considered the relative expression of CD11c [28, 29].

### Cytokine analysis

Sera were collected from the blood of all mice on day 45; the content of cytokines (TNF-a, IL-6, and IL-10) was measured by using the mouse magnetic Luminex screening assay (R&D Systems, Minneapolis, MN, USA).

### Analysis of DC phenotypes in the spleen

On day 45, spleen single-cell suspensions were collected from the five groups of mice, filtered with a cell strainer, and stained with PerCP/Cy5.5 anti-mouse CD11c, PE anti-mouse CD80, APC anti-mouse I-A/I-E (major histocompatibility complex II, or MHC II), or the corresponding isotype control (BioLegend, San Diego, CA, USA) for 25 min at 4 °C. After washing with wash buffer, cells were analyzed by using flow cytometry.

### Preparation of bone marrow–derived DCs

Bone marrow–derived DCs (BMDCs) were generated from the tibias and femurs of 6-week-old male C57BL/6 J mice as described previously [30, 31]. Cells were cultured in complete RPMI 1640 medium (HyClone, part of GE Healthcare, Chicago, IL, USA) supplemented with 10% fetal bovine serum, 20 ng/mL recombinant mouse granulocyte-macrophage colony-stimulating factor (rmGM-CSF), and 10 ng/mL rmIL-4 (PeproTech, Rocky Hill, NJ, USA) at a density of 5 × 106 cells/mL in six-well plates. After 1 day, non-adherent cells were washed off and new complete medium supplemented with 10% fetal bovine serum, 20 ng/mL rmGM-CSF, and 10 ng/mL rmIL-4 was added. New complete medium and cytokines were added every 3 days. On day 6 of culture, immature BMDCs were collected. Immature BMDCs were matured by further culturing in the presence of 1 μg/mL lipopolysaccharide (Sigma-Aldrich, Munich, Germany) for 24 h, and mature BMDCs were harvested.

### Phenotyping of BMDCs

BMDCs prepared as described earlier were harvested and stained with PerCP/Cy5.5 anti-mouse CD11c, PE anti-mouse CD80, APC anti-mouse I-A/I-E (MHC II), FITC anti-mouse F4/80, or the corresponding isotype control (BioLegend) for 25 min at 4 °C. After washing, cells were analyzed with flow cytometry. Data analysis was performed by using FlowJo software (Tree Star, Ashland, OR, USA), and the results were reported as mean fluorescence intensity.

### Statistical analysis

Statistical analysis was performed with GraphPad Prism software (GraphPad Software, La Jolla, CA, USA). Data were expressed as the mean ± standard deviation. Intergroup comparisons were performed by using one-way analysis of variance. If the data did not satisfy normal distribution, the rank sum test was used. A *P* value of less than 0.05 was considered significant.

## Results

### GTS-21 attenuates the inflammatory response in CIA mice

**Fig. 1:**
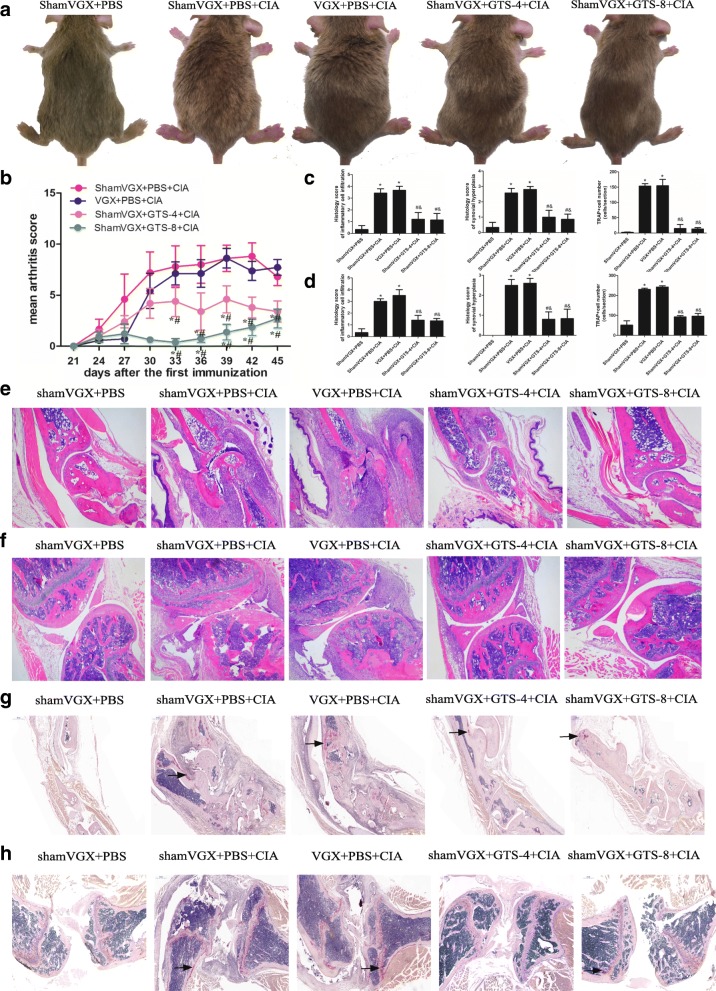
GTS-21 ameliorated inflammation in collagen-induced arthritis (CIA) mice. **a** The CIA model in DBA/1 mice was successfully established. GTS-21 decreased the redness and swelling of joints in CIA mice. **b** The arthritis score index was used to assess the severity of arthritis. GTS-21–treated CIA mice showed a dramatic decrease in arthritis scores compared with those in mice in the model group and vagotomy group. **c** and **d** The score of inflammatory cell infiltration (grade 0–4), synovial hyperplasia (grade 0–3), and tartrate-resistant acid phosphatase–positive (TRAP^+^) cells in the ankle (**c**) and knee (**d**). **e** and **f** GTS-21 decreased the infiltration of inflammatory cells and synovial proliferation in the ankle (e, 40×) and knee (f, 40×) in CIA mice. **g** and **h** GTS-21 decreased the infiltration of osteoclasts in the ankle (g, 40×) and knee (h, 40×) in CIA mice (the arrows point to TRAP^+^ cells). Data are expressed as the mean ± standard deviation (*n* = 8). *P <0.05 versus the model group; ^#^*P* <0.05 versus the vagotomy group

To determine whether the CAP regulates the inflammatory response in RA, the left vagus nerve was sectioned to inhibit the pathway, and GTS-21 was injected into the peritoneal cavity to activate the pathway 4 days before CIA induction. The degree of paw swelling was evaluated by using the arthritis index score every 3 days starting on day 21 after the first immunization. No paw swelling was observed in the control group, whereas the first signs of swelling appeared on day 24 in other groups. Joint swelling in the model and vagotomy groups reached a peak on days 42 and 39, respectively. The GTS-21 4 and 8 mg/kg groups showed mild joint swelling, and the arthritis scores were significantly lower than those in the model and vagotomy groups (*P* <0.05) (Fig. 1a and b).

The ameliorating effect of GTS-21 on CIA was confirmed by HE staining and TRAP staining of joints. The model and vagotomy groups showed infiltration of numerous inflammatory cells, osteoclasts, and synovial hyperplasia in the ankle and knee joints compared with the control group. The abnormalities were significantly alleviated in CIA mice after administration of GTS-21 (4 and 8 mg/kg) (Fig. 1c–h).

### GTS-21 reduces the levels of serum TNF-α and IL-6, but not IL-10, in CIA mice

**Fig. 2:**
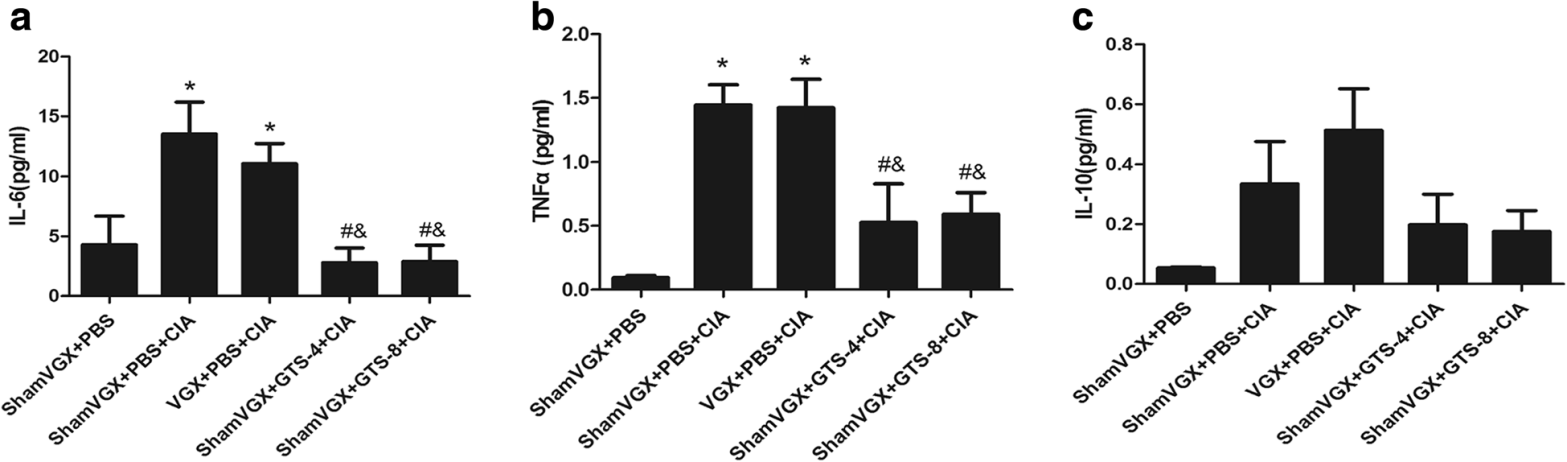
Analysis of cytokine levels in the serum of DBA/1 mice. On day 45 after the initial immunization, the serum was collected. Luminex was used to detect the levels of tumor necrosis factor-alpha (TNF-α), interleukin-6 (IL-6), and IL-10. GTS-21 reduced the levels of IL-6(**a**) and TNF-α(**b**) but had no effect on IL-10. Data are expressed as the mean ± standard deviation (n = 8). *P <0.05 versus the control group; ^#^*P* <0.05 versus the model group; ^&^*P* <0.05 versus the vagotomy group

To examine the effect of GTS-21 and vagotomy on inflammatory factors in the serum of DBA/1 mice, Luminex was used to detect the levels of IL-6, TNF-α, and IL-10. The serum levels of TNF-α and IL-6 were significantly higher in the model and vagotomy groups than in the control group, whereas GTS-21 (4 and 8 mg/kg) treatment markedly decreased the levels of these cytokines. There was no significant difference in the level of IL-10 between the five groups (Fig. 2a–c).

### GTS-21 downregulates the surface molecules CD80 and MHC II in DCs in the spleen of CIA mice

**Fig. 3:**
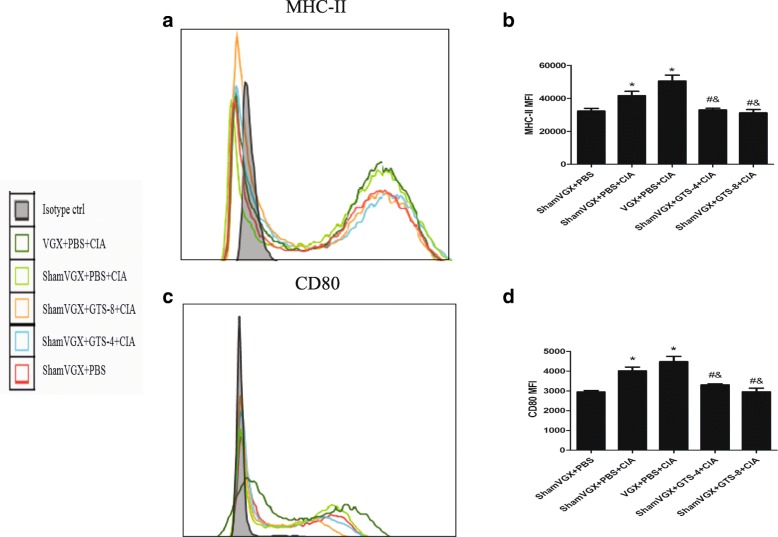
GTS-21 downregulated the expression of CD80 and major histocompatibility complex II (MHC II) on the surface of dendritic cells (DCs) in the spleen. **a**, **c** Histograms of MHC II and CD80 in CD11c^+^ cells are shown. **b**, **d** The changes of mean fluorescence intensity (MFI) of MHC II and CD80 were analyzed. Data are expressed as the mean ± standard deviation (n = 8). *P <0.05 versus the control group; ^#^*P* <0.05 versus the model group; ^&^*P* <0.05 versus the vagotomy group

On day 45 after the first immunization, DBA/1 mice were humanely killed, and spleen single-cell suspensions were prepared. The expression of the co-stimulatory molecule CD80 and the antigen-presenting molecule MHC II on CD11c^+^ DCs was detected by using flow cytometry. The expression of CD80 and MHC II in DCs in the spleen was significantly higher in the CIA group and vagotomy group than in the control group, and treatment with GTS-21 (4 and 8 mg/kg) significantly downregulated CD80 and MHC II expression compared with that in the model and vagotomy groups (Fig. 3).

### GTS-21 decreases DC infiltration into the synovium in CIA mice

**Fig. 4:**
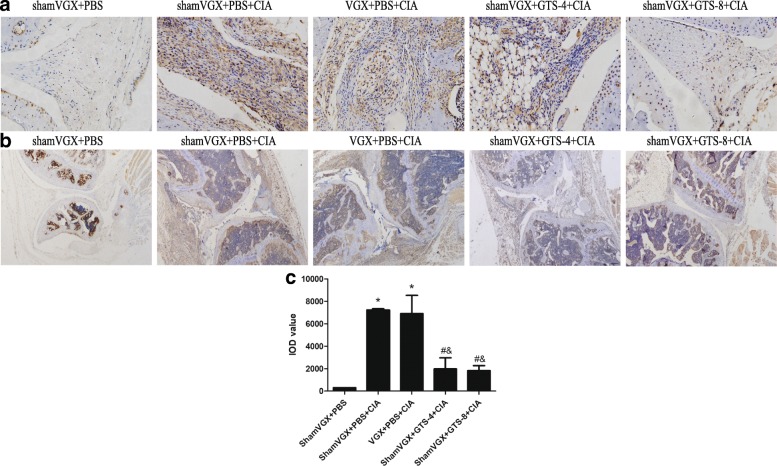
GTS-21 significantly downregulated CD11c expression in the synovium of collagen-induced arthritis (CIA) mice. **a**, **b** Immunohistochemical analysis was performed to detect CD11c expression in the knee joint tissues of mice (n = 8). CD11c^+^ cells (brown) were decreased significantly in the synovial tissues of the GTS-21 groups (a, 100×; b, 40×). **c** The integrated optical density (IOD) values of CD11c in synovial tissues were compared among the groups. Data are expressed as the mean ± standard deviation. *P <0.05 versus the control group; ^#^*P* <0.05 versus the model group; ^&^*P* <0.05 versus the vagotomy group

To further evaluate the effect of GTS-21 on DCs, the expression of the DC-specific marker CD11c was assessed in joint synovial tissues by immunohistochemistry. Few cells expressed CD11c in the control group. CD11c expression was significantly upregulated in the model and vagotomy groups. GTS-21 downregulated CD11c expression compared with that in the model and vagotomy groups (Fig. 4).

### GTS-21 inhibits BMDC differentiation

**Fig. 5:**
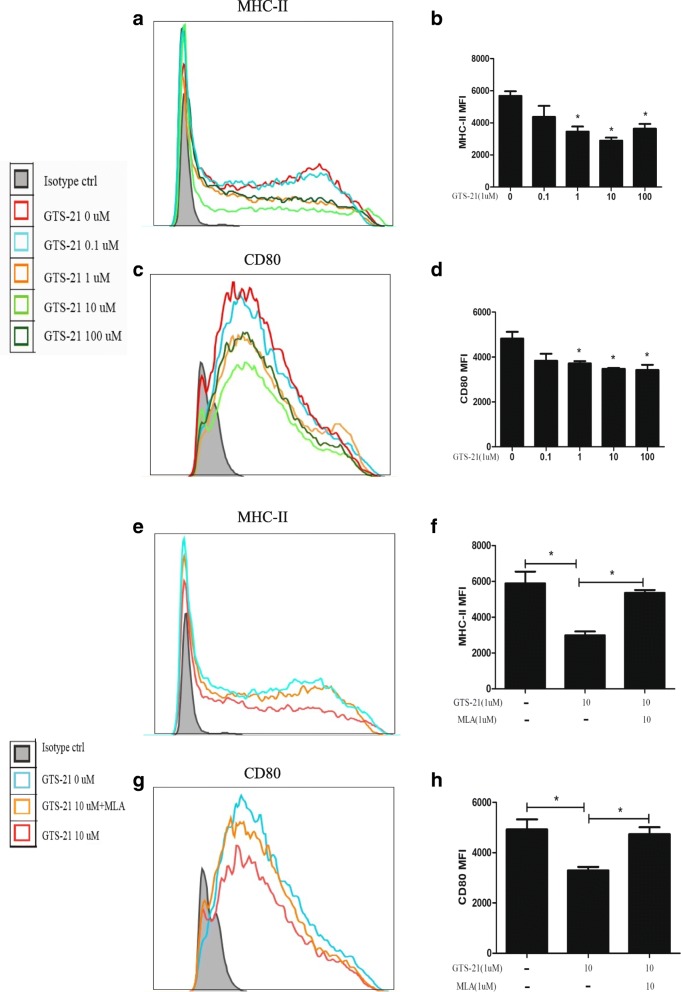
GTS-21 efficiently inhibited the expression of CD80 and major histocompatibility complex II (MHC II) on the surface of dendritic cells (DCs) during DC differentiation. Bone marrow–derived DCs were stimulated with GTS-21 (0.1, 1, 10, or 100 μmol/L) for 6 days. The expression levels of MHC II and CD80 were detected by using flow cytometry. All data shown were gated on CD11c^+^ F4/80^−^ cells. **a**, **c**, **e**, **g** Histograms of MHC II and CD80 in CD11c^+^ F4/80^−^ cells are shown. **b**, **d**, **f**, **h** The changes of mean fluorescence intensity (MFI) of MHC II and CD80 were analyzed. (b, d) GTS-21 (1, 10, or 100 μmol/L) decreased the MFI of CD80 and MHC II. (f, h) GTS-21 (10 μmol/L) significantly decreased the MFI of CD80 and MHC II, and methyllycaconitine (MLA) reversed this effect. Data are expressed as the mean ± standard deviation. *P <0.05 versus the control (“ctrl”) group. All data are representative of three independent experiments

To examine the direct effects of GTS-21 on DC differentiation, BMDCs were generated from C57BL/6 J mice. GTS-21 (0.1, 1, 10, or 100 μmol/L) was added to BMDCs (except the control group) cultured under DC differentiation conditions. CD11c is a relatively specific marker for BMDCs, and F4/80 is considered a macrophage marker [32]. To ensure that BMDCs and CD11c^+^ F4/80^−^ cells were gated (Additional file 1), within this population, the expression of CD80 and MHC II on DCs was measured with flow cytometry. The results showed that GTS-21 (1, 10, or 100 μmol/L) significantly inhibited the expression of the surface molecules MHC II (Fig. 5a and b) and CD80 (Fig. 5c and d) in DCs. The effect of GTS-21 (10 μmol/L) on inhibiting the expression of these molecules was significant and antagonized by the selective α7nAChR antagonist methyllycaconitine (MLA) (10 μmol/L) (Fig. 5e–h).

### GTS-21 has no obvious effect on BMDC maturation

**Fig. 6:**
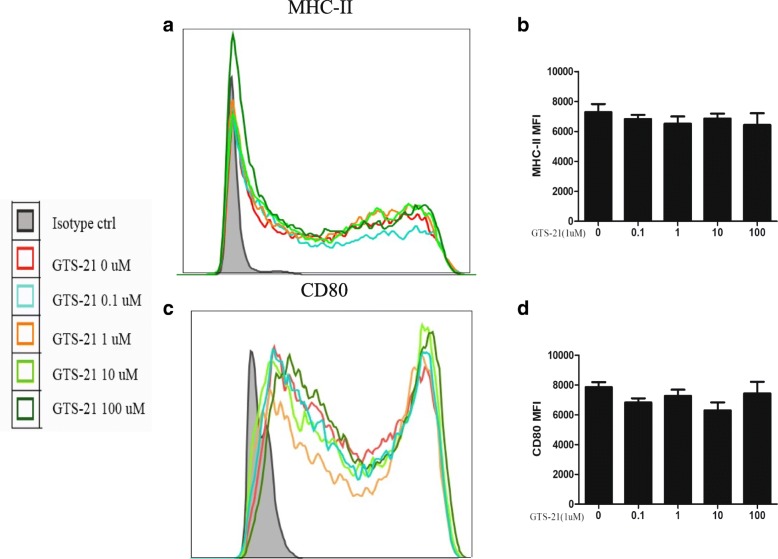
GTS-21 had no effect on the expression of CD80 and major histocompatibility complex II (MHC II) on the surface of dendritic cells (DCs) during DC maturation. On day 6 of bone marrow–derived dendritic cell culture, immature DCs were stimulated with GTS-21 (0.1, 1, 10, or 100 μmol/L) for 24 h. The expression levels of MHC II and CD80 were detected by using flow cytometry. All data shown were gated on CD11c^+^ F4/80^−^ cells. **a**, **c** Histograms of MHC II and CD80 in the CD11c^+^ F4/80^−^ cells are shown. **b**, **d** The changes of mean fluorescence intensity (MFI) of MHC II and CD80 were analyzed. Data are expressed as the mean ± standard deviation. All data are representative of three independent experiments. Abbreviation: *ctrl* control

To induce BMDC maturation, lipopolysaccharide (LPS) (1 μg/mL) was added to BMDCs cultured under differentiation conditions on day 6. GTS-21 (0.1, 1, 10, or 100 μmol/L) was added to inhibit the maturation process. The expression of CD80 and MHC II on BMDCs was detected with flow cytometry on day 7. Compared with the BMDCs on day 6, LPS upregulated the expression of the surface molecules CD80 and MHC II, and GTS-21 had no obvious effect on CD80 and MHC II expression (Fig. 6).

## Discussions

DCs are potent antigen-presenting cells that play a major role in the regulation of immune responses in RA. Rheumatoid synovial fluid and synovial tissues are enriched in mature DCs, which participate in the inflammatory cascade by secreting specific T cell–attracting chemokines and through the ongoing presentation of antigen to autoreactive T cells [33–35]. In animal models, administration of collagen-pulsed mature DCs is sufficient to induce arthritis [36]. This suggests that DCs are a valuable target for the management of RA. Here, we explored whether the anti-inflammatory pathway can prevent the development of RA through the modulation of DCs.

First, we investigated the effects of the cholinergic agonist GTS-21 on the pathogenesis of RA in a CIA model. Activation of the CAP with GTS-21 markedly reduced clinical arthritis, inflammatory cell infiltration, synovial hyperplasia, and bone damage. TNF-α and IL-6 are key pro-inflammatory cytokines in RA [37, 38]. Therefore, TNFα and IL-6 levels were detected in the serum of CIA mice. The results showed that treatment with GTS-21 decreased the levels of the two cytokines. These data indicated that the CAP exerted strong anti-inflammatory effects in CIA mice. However, vagotomy did not exacerbate the inflammation in CIA mice, indicating that the contralateral vagus nerve may have a compensatory role. Further study is necessary to clarify this issue [19, 21]. Moreover, a study showed that denervation protected limbs from arthritis using the K/BxN serum-transfer system by affecting the microvasculature [39]. More research is needed to explore the link between the nervous and immune systems.

The immunologic mechanism underlying the effect of the CAP on protecting against CIA remains unclear. The results of this study demonstrated that the expression of the co-stimulatory molecules CD80 and MHC II in CD11c^+^ DCs in the spleen was upregulated in the CIA group, which is consistent with the results of a previous study [40]. We confirmed that DCs are involved in inflammation associated with CIA. GTS-21, a selective α7nAChR agonist, has been used in clinical trials and is less toxic than nicotine [41, 42]. GTS-21 (4 mg/kg) significantly improves survival in murine models of endotoxemia, severe sepsis, and burns [43, 44]. Our data firstly confirmed the immunomodulatory effects of GTS-21 on DCs in CIA mice. GTS-21 (4 and 8 mg/kg) significantly downregulated the expression of MHC II and CD80 on the surface of DCs in the spleen of CIA mice. CD80 and MHC II are important surface molecules involved in the activation of Ag-specific CD4^+^ T cells [40], which suggests that the anti-inflammatory activity of the CAP in RA may be mediated, at least in part, by the modulation of DCs. CD11c is a relatively specific marker of DCs in mice [45, 46]. The present results showed that CD11c was upregulated in the joint synovium of CIA mice and that GTS-21 treatment downregulated CD11c. This suggests that the anti-inflammatory pathway can directly affect the infiltration of DCs into the synovium.

DCs are derived from hematopoietic stem cells or peripheral blood mononuclear cells [47, 48]. The expression of related specific markers such as HLA-DR on the surface of DCs increases during differentiation [49]. Immature DCs have a strong capacity for antigen uptake. Once activated, DCs are converted into mature DCs, which express high levels of the co-stimulatory molecules CD80/86 and the antigen-presenting molecule MHC II, and stimulate T-cell proliferation [50–52]. To determine whether GTS-21 suppressed the infiltration of DCs into the synovium by affecting DC differentiation or maturation, we performed a follow-up experiment. BMDCs were generated from mouse bone marrow progenitor cells through stimulation with GM-CSF and IL-4 *in vitro* [53], and LPS can induce the mutation of BMDCs. Our results showed that GTS-21 inhibited the differentiation of BMDCs from progenitor cells but had no effect on the maturation of BMDCs, which indicated that GTS-21 exerts an anti-inflammatory effect by inhibiting the differentiation of DCs. The effects of GTS-21 on BMDC differentiation were counteracted by the acetylcholine receptor antagonist MLA, confirming that GTS-21 affected DC differentiation by activating the α7nAchR.

The mechanism underlying the suppression of DCs by GTS-21 remains unclear. GM-CSF, a critical factor for DC development, can target multiple intracellular signaling pathways to affect DC differentiation, including the Janus kinase/signal transducer and activator of transcription (JAK/STAT) pathway, the mitogen-activated protein kinase (MAPK) pathway, and the phosphatidylinositol 3-kinase (PI3K) pathway [54]. Inhibition of JAK2/STAT5, among these pathways, suppresses terminal DC differentiation [55, 56]. The MAPK and nuclear factor kappa-light-chain-enhancer of activated B cells (NF-κB) signaling pathways are involved in the maturation of DCs. Further experiments are needed to determine whether GTS-21 can regulate GM-CSF–related DC differentiation pathways.

## Conclusions

In summary, our research first investigated the anti-inflammatory effect of the cholinergic agonist GTS-21 on DCs in CIA. The present data indicated that GTS-21–mediated activation of the CAP inhibited DC differentiation and ameliorated inflammation in a CIA model. These results may provide new insight into the immune regulatory mechanism underlying the activity of the CAP in RA.

## Additional file

Additional file 1: Bone marrow–derived dendritic cells (BMDCs) were stained with antibodies against F4/80, CD11c, CD80 and major histocompatibility complex II (MHC II) and analyzed by flow cytometry. Frequency of CD11c^+^ F4/80^−^ cells was analyzed by flow cytometry. The number in the upper right represents the percentage of CD11c^+^ F4/80^+^ cells, which is less than 5%. (DOCX 50 kb)

## Abbreviations

α7nAChR: α7 subunit of the nicotinic acetylcholine receptor
BMDC: Bone marrow–derived dendritic cell
CAP: Cholinergic anti-inflammatory pathway
CIA: Collagen-induced arthritis
DC: Dendritic cell
GM-CSF: Granulocyte-macrophage colony-stimulating factor
IL: Interleukin
IOD: Integrated optical density
JAK/STAT: Janus kinase/signal transducer and activator of transcription
LPS: Lipopolysaccharide
MAPK: Mitogen-activated protein kinase
MHC II: Major histocompatibility complex II
MLA: Methyllycaconitine
PBS: Phosphate-buffered saline
RA: Rheumatoid arthritis
rmGM-CSF: Recombinant mouse granulocyte-macrophage colony-stimulating factor
TNFα: Tumor necrosis factor-alpha
TRAP: Tartrate-resistant acid phosphatase
VGX: Vagotomy
