# Supplementary material for: The effect of the cholinergic anti-inflammatory pathway on collagen-induced arthritis involves the modulation of dendritic cell differentiation
Source: Arthritis Res Ther. 2018 Nov 28;20:263. doi: 10.1186/s13075-018-1759-9 (PMC6262974; doi:10.1186/s13075-018-1759-9)
Supplement: Supplementary file 1 — Bone marrow–derived dendritic cells (BMDCs) were stained with antibodies against F4/80, CD11c, CD80 and major histocompatibility complex II (MHC II) and analyzed by flow cytometry. Frequency of CD11c+ F4/80− cells was analyzed by flow cytometry. The number in the upper right represents the percentage of CD11c+ F4/80+ cells, which is less than 5%. (DOCX 50 kb) [file 13075_2018_1759_MOESM1_ESM.docx]

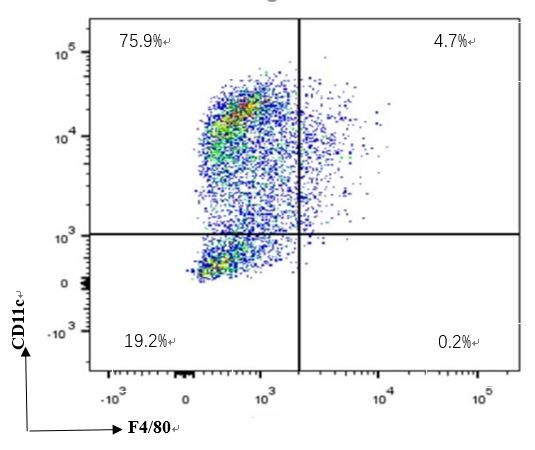


Figure S1

BMDCs were stained with antibodies against F4/80, CD11c, CD80 and MHC-II and analyzed by flow cytometry . Frequency of CD11c+F4/80- cells was analyzed by flow cytometry. The number in the upper right represents the percentage of CD11c+ F4/80+cells, which is less than 5%.
